# Pirfenidone and Nintedanib in Pulmonary Fibrosis: Lights and Shadows

**DOI:** 10.3390/ph17060709

**Published:** 2024-05-30

**Authors:** Maria Chianese, Gianluca Screm, Francesco Salton, Paola Confalonieri, Liliana Trotta, Mariangela Barbieri, Luca Ruggero, Marco Mari, Nicolò Reccardini, Pietro Geri, Michael Hughes, Selene Lerda, Marco Confalonieri, Lucrezia Mondini, Barbara Ruaro

**Affiliations:** 1 Pulmonology Unit, Department of Medical Surgical and Health Sciences, University of Trieste, Hospital of Cattinara, 34149 Trieste, Italy; maria.chianese1989@gmail.com (M.C.);; 2 Division of Musculoskeletal and Dermatological Sciences, Faculty of Biology, Medicine and Health, The University of Manchester & Salford Royal NHS Foundation Trust, Manchester M6 8HD, UK; 3 Graduate School, University of Milan, 20149 Milano, Italy

**Keywords:** idiopathic pulmonary fibrosis, Nintedanib, Pirfenidone, interstitial lung disease, forced vital capacity

## Abstract

Pirfenidone and Nintedanib are specific drugs used against idiopathic pulmonary fibrosis (IPF) that showed efficacy in non-IPF fibrosing interstitial lung diseases (ILD). Both drugs have side effects that affect patients in different ways and have different levels of severity, making treatment even more challenging for patients and clinicians. The present review aims to assess the effectiveness and potential complications of Pirfenidone and Nintedanib treatment regimens across various ILD diseases. A detailed search was performed in relevant articles published between 2018 and 2023 listed in PubMed, UpToDate, Google Scholar, and ResearchGate, supplemented with manual research. The following keywords were searched in the databases in all possible combinations: Nintedanib; Pirfenidone, interstitial lung disease, and idiopathic pulmonary fibrosis. The most widely accepted method for evaluating the progression of ILD is through the decline in forced vital capacity (FVC), as determined by respiratory function tests. Specifically, a decrease in FVC over a 6–12-month period correlates directly with increased mortality rates. Antifibrotic drugs Pirfenidone and Nintedanib have been extensively validated; however, some patients reported several side effects, predominantly gastrointestinal symptoms (such as diarrhea, dyspepsia, and vomiting), as well as photosensitivity and skin rashes, particularly associated with Pirfenidone. In cases where the side effects are extremely severe and are more threatening than the disease itself, the treatment has to be discontinued. However, further research is needed to optimize the use of antifibrotic agents in patients with PF-ILDs, which could slow disease progression and decrease all-cause mortality. Finally, other studies are requested to establish the treatments that can stop ILD progression.

## 1. Introduction

Interstitial lung diseases (ILDs) comprise nearly a fifth of all lung diseases. ILDs are a heterogeneous group of disorders classified into distinct entities, each with specific clinical, radiological, and pathological characteristics [1,2,3,4,5,6,7,8]. Most ILDs are characterized by a progressive distortion of airway architecture, leading to reduced lung volume and gradual loss of lung function, which can eventually result in respiratory failure [2,3,4,5,6,7,8,9].

Idiopathic pulmonary fibrosis (IPF) accounts for about 20% of ILDs, although once considered a rare disorder, its incidence and prevalence are increasing globally. The world’s highest prevalence occurs in South Korea with 4.51 cases per 10,000 persons, whereas in Europe, according to the most recent evidence in the literature, the estimated prevalence of IPF is estimated to be between 0.33 and 2.51 per 10,000 inhabitants [3,10,11,12,13,14,15,16]. The prevalence is estimated to be higher in men than in women, with a median age of 60 years old [4,17,18,19,20,21,22,23,24,25].

**Figure 1:**
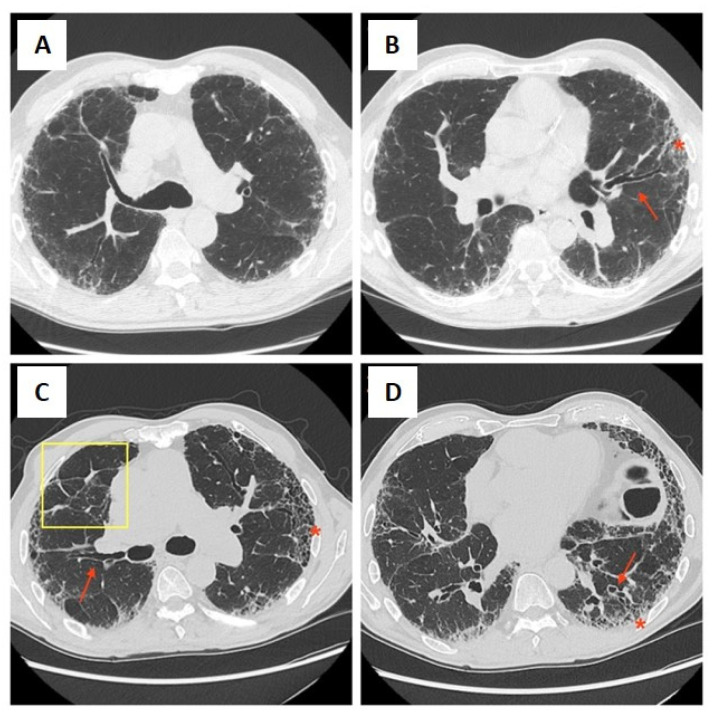
CT lung scan of a 75-year-old male patient at the time of UIP diagnosis at our center, after Pirfenidone was started. The CT scan in boxes (**A**,**B**) show traction bronchiectasis (red arrow) and honeycombing areas mainly located at the basal zone (red asterisk). CT images in boxes (**C**,**D**) were acquired after 3 years of follow up and specific therapy and show progression in findings already present (red arrow and asterisk) together with septal thickening (yellow square), another UIP radiological feature.

IPF is a chronic, progressive fibrosing interstitial pneumonia of unknown cause, characterized by the progressive worsening of lung function and physiological impairment associated with a poor prognosis, and it shares radiographic pattern of usual interstitial pneumonia (UIP), as shown in Figure 1.

Several risk factors are implicated in pathogenesis of idiopathic pulmonary fibrosis, including environmental factors, smoking, viral infections, and other comorbidities. When the lung is exposed to innumerable injuries, it enacts a series of repair and recovery processes that are finely synchronized with each other. However, in some cases, especially in predisposed individuals, continuous alveolar epithelial injury can lead to several consequences, including profibrotic epigenetic reprogramming, premature senescence, excessive production of profibrotic factors, and the activation of mesenchymal cells, which could lead to the development of pulmonary fibrosis [5,26,27,28,29]. The fibrosis process that characterizes ILDs and the associated hypoxemia led to pulmonary vasoconstriction and a reduction in angiogenesis through various pathological mechanisms, which result in, among other things, a reduction in capillary density. These conditions, secondary to a state of chronic inflammation, increase the risk of developing pulmonary hypertension (PH), which is part of the natural history of patients with ILD. This process is particularly pronounced in patients with SC-ILD, where the vasculitic aspect is predominant. The combination of interstitial pathologies and PH thus leads to a further worsening at the level of the alveolus–capillary membrane, which results in a deterioration of gas exchange, and consequently, lung function, which results in a clinical worsening of dyspnoea [30,31,32]. 

IPF is a chronic and complex lung disease whose pathophysiology is not yet fully elucidated, which is why the management of these patients is difficult and mainly focused on improving health status, preserving lung function, and, ideally, ameliorating survival and quality of life [31,32,33,34,35,36].

To date, the pharmacological options for the treatment of IPF are Nintedanib and Pirfenidone, two drugs designed to slow disease progression that have shown to have similar effects in real life [6,7,31,32,33,34,35,36].

Nintedanib acts as a triple tyrosine kinase inhibitor (TKI) targeting the receptor kinases of platelet-derived growth factor (PDGF), fibroblast growth factor (FGF), and vascular endothelial growth factor (VEGF). These receptors play a crucial role in IPF pathogenesis by inhibiting the proliferation and activation of human fibroblast, reducing the release of profibrotic and inflammatory mediators, decreasing extracellular matrix deposition, and inhibiting vessel proliferation [8,35]. Its antifibrotic and anti-inflammatory activities have been demonstrated in multiple in vitro assays and animal models of lung fibrosis [37,38,39,40,41,42,43]. A 2019 in vitro and in vivo study also examined the impact of Nintedanib on pulmonary hypertension (PH), elucidating its mechanisms of action. The study demonstrated that this medication enhances hemodynamics and mitigates vascular remodeling, including reductions in neointimal lesions and medial wall thickening, thereby displaying anti-vascular remodeling effects. 

Pirfenidone (5-methyl-1-phenyl-2-[1H]-pyridone) acts by regulating tumor necrosis factor (TNF) and transforming growth factor-β (TGF-β) pathways, inhibiting fibroblast proliferation and collagen deposition. These mechanisms explain its antifibrotic and anti-inflammatory effects [9,10,11], which have been validated in various in vitro and in vivo trials. 

Moreover, Pirfenidone has been demonstrated to have a significant statistical impact on the decline of forced vital capacity (FVC), and it is better than other antifibrotic drugs [9,10]; so, it was approved for the treatment of IPF in Europe in 2011 and in the USA in 2014. 

The introduction of these two pharmacological options has revolutionized ILD management. Both drugs have demonstrated the ability to slow disease progression in terms of lung function and reduce the rate of hospitalizations and associated mortality due to respiratory diseases. However, to date, the impact of antifibrotic drugs appears to be less significant on patients’ symptoms, and in some cases, they are poorly tolerated. Therefore, the approach to ILD patients must be multimodal, incorporating not only antifibrotic drugs but also symptom management through rehabilitation, addressing risk factors and comorbidities, and providing patient support. Additionally, involvement in clinical trials could be beneficial.

The main objective of this work is to review recent advances in the management of fibrosing ILD, focusing, in particular, on IPF, post-COVID-19 fibrosis, interstitial lung disease associated with systemic sclerosis (SSc-ILD), ILD associated with rheumatoid arthritis (RA-ILD), idiopathic inflammatory myopathies (IIM)-ILD, and stage IV sarcoidosis by comparing the two main antifibrotic drugs and assessing any difference in efficacy on fibrosis progression, tolerability, and use in clinical practice.

## 2. Pirfenidone and Nintedanib in ILDs

Antifibrosant drugs are widely used in IPF and SSc-ILD; beyond this pathology, we deepened their therapeutic use in post-COVID-19 fibrosing interstitial disease, rheumatoid arthritis, and sarcoidosis with lung involvement and, finally, in idiopathic inflammatory myopathies (IIM)-ILD [32,33,34].

Many clinical trials have proven the efficacy of Pirfenidone in IPF. The multinational randomized pivotal trial CAPACITY and ASCEND found that Pirfenidone reduces the decline of forced vital capacity (FVC), which reflects a slowing down of disease progression, an increased six-minute walking test (6MWT) distance, and higher progression-free survival compared to placebo [29,31]. 

This is related to a reduced risk of death since the decline in FVC is validated as the most important predictor of mortality in patients with IPF [32], and no significant effect on acute exacerbation of IPF was nevertheless demonstrated in these studies [33].

Pirfenidone allows the tapering or the interruption of other therapies, such as corticosteroids or immunosuppressive agents [34]. 

Regarding Nintedanib, two pivotal, phase 3 randomized controlled trials, the INPULSIS-1 and INPULSIS-2, showed a reduced decline in FVC in patients treated with 150 mg of Nintedanib twice daily compared to placebo over 52 weeks of treatment, which is related with a reduced progression of the disease. Although, no relevant modifications were observed in the number of exacerbations or on the St. George’s Respiratory Questionnaire (SGRQ) [38]. 

These findings were later confirmed by real-life studies [39,40,41,42,43,44,45].

Few comparative studies between the two drugs were performed, and they showed no statistically significant differences [42,43,44,45,46]. 

Even if the use of Pirfenidone and Nintedanib slows down the progression of the disease and reduces all-cause mortality in long-term studies [26,47], IPF has a progressive and fatal outcome, and further research is required. The future possibility of treatment could be an association of the two molecules because they act on different pathogenic mechanisms in fibrogenesis, and this could lead to an improvement of the outcome compared to the single therapy [47]. The risk of combining the two drugs could be the increase in adverse events; but, in recent studies conducted on combination therapies, these effects were manageable [47,48]. 

**Figure 2:**
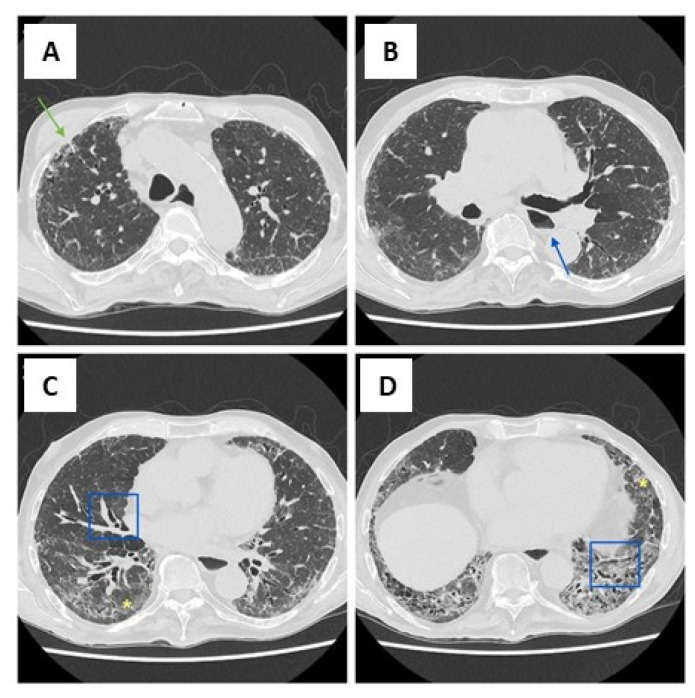
Radiological images of a 72-year-old man with SSC-ILD (**A**–**D**). CT scans show the main features of NSIP, which is the most common pattern in patients with systemic sclerosis. In particular, traction bronchiectasis (in the blue square) (**C**,**D**), ground glass areas (yellow asterisk) (**C**,**D**) with a symmetrical distribution and mainly peripheral and lower lobe involvement, and reticular opacities (green arrow) (**A**) are noted. The blue arrow also indicates the dilated esophagus (**B**), another feature observed in patients with systemic sclerosis.

Systemic sclerosis (SSc) is a connective tissue disease (CTD) that affects multiple organs, including skin, blood vessels, heart, lungs (Figure 2), kidneys, and the gastrointestinal system. It is associated with significant morbidity and mortality, primarily due to fibrosis and vasculopathy. ILD is a common manifestation of SSc and a leading cause of related death.

According to the latest European Alliance of Associations for Rheumatology (EULAR) guidelines, the treatment of SSc-ILD is based on the immunosuppressants mycophenolate (MMF) and Cyclophosphamide (CYC). The American Thoracic Society (ATS) guidelines have also recently expressed a therapeutic approach for SC-ILDs, agreeing on the use of mycophenolate and expressing conditional favor regarding Cyclophosmide and Nintedanib, possibly in combination with mycophenolate. With regard to Pirfenidone, however, it was pointed out that further research is needed [49,50,51,52]. 

The well-known “INBUILD” study established that in patients with progressive fibrosing interstitial lung diseases, including SSc-ILD, the annual rate of decline in FVC was significantly lower among patients who received Nintedanib compared to those who received placebo [53]. This was confirmed by the SENSCIS study that demonstrated an annual rate of change in FVC of −52.4 mL per year in patients treated with Nintedanib compared with −93.3 mL in the placebo group (difference, 41.0 mL per year; 95% confidence interval [CI], 2.9 to 79.0; *p* = 0.04) [54]. 

A subgroup analysis of the SENSCIS study highlighted that a combination of MMF and Nintedanib could offer a safe therapeutic option for patients with SSc-ILD, with a similar tolerability profile compared to MMF alone. However, further data are necessary to demonstrate the effective benefit of the initial combined therapy compared to a sequential approach [55]. Several studies have demonstrated the effectiveness of the drug for up to 100 weeks. Regarding tolerability, the occurrence of gastrointestinal side effects in patients treated with Nintedanib is frequent, and dose reductions or interruptions were more frequent in female patients, who are known to be the most affected by SSc [55]. 

Finally, Nintedanib showed no particular effect in terms of efficacy on dermatological lesions, as observed by the change from the baseline in the modified Rodnan skin score, and the total score on the SGRQ at week 52 did not differ significantly between the study groups, with differences of −0.21 (95% CI, −0.94 to 0.53; *p* = 0.58) and 1.69 (95% CI, −0.73 to 4.12 [unadjusted for multiple comparisons]), respectively [53].

We can affirm that Nintedanib could be a valid therapeutic opportunity in SSc-ILD, especially in progressive pulmonary fibrosis (PPF). However, further data are needed to justify its use in the initial stages of the disease, especially by carrying out a careful risk–benefit ratio regarding the onset of side effects. 

Despite having distinct triggers, both IPF and SSc-ILD share common pathophysiological processes, involving the conversion of fibroblasts into a myofibroblastic phenotype and the excessive accumulation of extracellular matrix, a process that Nintedanib can regulate according to its mechanism of action [53,54,55]. 

Pirfenidone has received less comprehensive evaluation in SSc-ILD; however, there are still studies that have assessed its efficacy and safety profile.

The LOTUSS clinical study highlighted that the drug is safe and well tolerated, even when combined with MMF. In fact, although most patients developed Treatment-Emergent Adverse Events, these rarely caused a suspension of the drug, especially when the titration of the drug occurred in 4 weeks instead of 2 [56]. The RELIEF study, an RCT on progressive fibrotic ILD that also included patients with SSc, showed that Pirfenidone slows the course of the disease, especially in relation to the decline of FVC. Unfortunately, this study was stopped due to slow recruitment [57]. 

The Scleroderma Lung Study III (SLS III) trial compared the efficacy of the combination of MMF and Pirfenidone versus MMF alone as active therapy for scleroderma-related ILDs. This study was discontinued because it failed to involve the intended number of participants, having only enrolled 51 out of the targeted 150 patients; therefore, the results are only partially interpretable, and further studies are necessary. Nevertheless, no difference in overall improvement was found between MMF and MMF plus Pirfenidone at 18 months. However, there was a more rapid improvement in the 6-month predicted FVC rate, patient-reported outcomes, and High-Resolution Computed Tomography (HRCT) in the combination group [58]. In patients with scleroderma, where the underlying pathology may already involve skin manifestations, the dermatological side effects of Pirfenidone become even more significant and may pose a greater challenge in managing side effects. At present, available data and the small number of patients who have been tested with this drug do not allow us to endorse its use in patients with Sc-ILD. As the guidelines also indicate, future studies with larger sample sizes are necessary to provide a favorable opinion on this therapy. 

SARS-CoV-2 infection can result in post-acute-phase sequelae (PASC), causing a disruption of alveolar architecture characterized by collagen fiber deposition and increased cellular activity, ultimately leading to fibrosis formation. Moreover, it has been estimated that the prevalence of post-COVID-19 fibrosis is approximately 30 times higher than that of IPF [59,60,61]. However, it must be taken into account that these data refer to the sequelae of a disease declared a pandemic by the World Health Organization (WHO) in March 2020. The significant activation of fibroblasts in pulmonary fibrosis depends on the activation of various pathways, such as the signal transducer and activator of transcription 3 (STAT3), reactive oxygen species (ROS), and Interleukin-6 (IL6) on TGFβ [62]. Elevated plasma concentrations of VEGF, PDGF, and FGF have been observed in COVID-19 patients, suggesting the potential effectiveness of Nintedanib. Moreover, this agent has been shown to downregulate the expression of IL-1 and IL-6, pivotal cytokines in the COVID-19 cytokine storm associated with lung fibrogenesis. In the meantime, environmental factors and patient-related comorbidities are also connected to the development of post-COVID-19 fibrosis, such as a history of smoking, age, hospitalization, prolonged stay in an intensive care unit, mechanical ventilation, and chronic alcoholism [63]. Pirfenidone and Nintedanib have been studied as therapies for the treatment of fibrosis after SARS-CoV-2 infection. Available studies are observational and conducted on small samples, including case series and reports. They report the efficacy of PASC early lung sequelae, and most of them have not been examined in the recent review by Alrajhi addressing post-COVID-ILD, which highlighted the scarcity of high-quality data [64,65,66,67,68,69,70,71,72]. Sahajal Dhooria et al. conducted a multicenter, retrospective survey study of subjects administered Pirfenidone or Nintedanib for post-COVID-19 interstitial lung abnormalities. Antifibrosant drugs were prescribed in 2% of patients, and 70% of them had significant or partial improvement in lung abnormalities on radiology. The authors suggested the need for larger studies with case–control groups to understand the actual usefulness of antifibrosants in this type of pathology [73]. Among the various case reports considered, Bussolari et al. discussed three cases of patients suffering from respiratory failure due to SARS-CoV-2 infection requiring orotracheal intubation with the radiological development of interstitial lung disease. Throughout hospitalization, Nintedanib was administered at its maximum dosage, and a gradual improvement in radiological findings of interstitial disease was observed, indicating a potential role for Nintedanib in modulating lung inflammation and promoting healing [71]. 

Ogata et al. reported on a patient with post-COVID-19 fibrosis treated with both steroids and Nintedanib who had reduced their oxygen support from high flow to 4 L/min after three months of therapy [66]. In a prospective study conducted by Buğra Kerget et al., 30 patients were randomized in a 1:1 ratio to receive either Nintedanib or Pirfenidone. Both treatment groups showed improvement, with Nintedanib demonstrating a more favorable response. The assessment of improvement included parameters such as pulmonary function tests, 6 min walk test (6MWT), peripheral oxygen saturation, and radiological CT scans [66].

However, the actual effectiveness of the two drugs requires further studies and demonstrations that, in fact, some molecular aspects of post-COVID-19 fibrosis are not completely explained by the phlogistic aspect of the infection alone [74]. Furthermore, a retrospective observational study conducted by Narongkorn Saiphoklang et al. showed that Nintedanib in post-COVID-19 fibrosis did not improve outcomes, such as 60-day mortality or radiographic findings, but led to an improvement in the SpO2/FiO2 ratio, concluding that further studies are necessary before the use of antifibrotic therapy [75]. In this regard, there are already several studies concerning the role of both Pirfenidone (FIBRO-COVID, NCT04607928) and Nintedanib (NINTECOR, NCT04541680) for PCILD. Finally, the PINCER trial (NCT04856111) will compare the safety and efficacy of these established antifibrotic treatments in a phase 4 study. 

Antifibrotic agents in stage IV sarcoidosis are an area of ongoing research [76]. Pirfenidone efficacy in sarcoidosis with lung fibrosis is currently being studied in a clinical trial [77]. Nintedanib efficacy in sarcoidosis patients is evaluated for 52 weeks on Nintedanib vs. placebo. Nintedanib use was associated with reduced ILD progression, measured with a decline in predicted FVC > 10% (HR 0.66 [95% CI: 0.53, 0.83]; *p* = 0.0003 [78,79]), so these results are promising for its use.

Nintedanib has also been tested in patients with RA-ILD [80,81,82,83,84,85,86]. The use of Nintedanib in patients with RA-ILD may be eligible as a potential additional treatment to improve the outcomes. The role of Nintedanib in improving the respiratory functions in these patients is proven by many articles. 

A recent study has established a correlation between Nintedanib and significant improvements in DLCO parameters, suggesting the potential benefit of initiating Nintedanib therapy early in addition to standard treatments [81]. In addition, a study was conducted in an SGK mouse model, in which autoimmune disease was induced with zymosan, which showed that early Nintedanib administration effectively reduced joint swelling and arthritis scores compared to a placebo cohort [82]. 

On the other hand, Pirfenidone has shown both antifibrotic effects due to its ability to manage oxidative stress by mainly reducing toxic hydroxyl radicals [83], and antirheumatic effects thanks to the inhibition of pro-inflammatory cytokines and the reduction in joint swelling, as seen in mice with induced arthritis treated with Pirfenidone [84]. Moreover, the promising role of Pirfenidone in RA-ILD needs to be further investigated by many clinical trials. TRAIL-1 was a quite promising study with the aim of investigating the efficacy of Pirfenidone administration in patients with RA-ILD, but unfortunately, it was stopped due to the COVID-19 outbreak, even if initial data were promising since Pirfenidone has not just slowed down the FVC decline over the time but also no new side effects were detected, and its co-administration with DMARD therapy did not reveal any relevant interactions [85]. These Pirfenidone-favorable effects were likewise described in another similar trial [57]. In another study, the efficacy and safety profile of Pirfenidone, when used in combination with standard therapy, were evaluated in patients with connective tissue disease-associated interstitial lung disease. The study compared the outcomes of the combination of standard therapy and Pirfenidone with those of a control group receiving standard therapy alone [86]. Patients with RA-ILD who underwent Pirfenidone treatment responded with a significant increase in DLCO% by 7.4% and improved FVC%, while in the control group, DLCO% decreased by 5.5%, suggesting an enhanced pulmonary function in the test group rather than in the control group [87]. 

The role of antifibrotic drugs in IIM-ILD has never been defined or assessed. Their potentials have never been studied in large trials; however, the scientific literature presents a smaller series that demonstrated the efficacy of both Pirfenidone and Nintedanib in (IIM)-ILD. 

For the efficacy of Pirfenidone, a prospective study enrolled 27 patients with early ILD (less than 6 months of disease duration) in amyopathic dermatomyositis (CADM) and administered them Pirfenidone, in addition to standard immunosuppression therapy. This cohort has been compared to an early retrospective study, where patients did not undergo Pirfenidone treatment. With the administration of Pirfenidone, a considerable reduction in the mortality rates in patients with subacute ILD (duration ranging from 3 to 6 months) has been shown. Nevertheless, in patients with acute ILD (less than 3 months of disease duration), there were no significant variations in survival benefit. Radiographic images in patients with Pirfenidone treatment were basically the same as those of patients with traditional therapy, and no FVC differences were able to be assessed by the authors due to a lack of data. To date, three survivors had to discontinue Pirfenidone due to ARDs [87]. 

Furthermore, a retrospective study evaluated the possible role of Nintedanib in (IIM)-ILD and compared 36 patients who underwent Nintedanib administration, in addition to immunosuppression treatment in 115 patients with only immunosuppression therapy. Patients with Nintedanib treatment showed improvement in the probability of survival, and the drug also held back the development of rapidly progressive interstitial lung disease (RP-ILD). Concerning FVC and DLCO, no differences were detected in either group [88].

## 3. Tolerance of Antifibrotic Drugs in Real Clinical Practice

Antifibrotic therapy generally causes multiple adverse effects [89,90,91,92,93,94,95,96,97]. Gastrointestinal side effects were the most common and are the main reason for the discontinuation of antifibrotic therapy, especially Nintedanib [98,99,100,101,102,103]. 

In order to ensure adherence to antifibrosant therapy, current guidelines suggest active management and possibly temporary dosage reduction or discontinuation [12]. In particular, the recommended daily dose of Pirfenidone is 2403 mg/day, divided into three doses. Normally one-third of the dosage is started and gradually increased until the effective dose is reached; in the event of side effects, the dose may be temporarily reduced or discontinued. The same applies to Nintedanib, whose recommended dose is 300 mg/die (150 mg every 12 h), which may possibly be reduced to 100 mg twice in the event of uncontrolled side effects. 

Several studies regarding Nintedanib adverse events (AEs) described gastrointestinal disorders, including diarrhea and nausea, as more frequent, occurring in over two-thirds of cases within the first 3 months of therapy with mild to moderate intensity; however, these are manageable with specific therapy (particularly antiemetics and anti-diarrhoeic) and behavioral modification [18,19,24].

Nintedanib was only discontinued in a low percentage of patients (from 10% to 26%, according to several studies) due to intolerance to and uncontrollability of AEs [20,26,27]. Furthermore, alterations in liver function parameters have been described, with ALT and/or AST values ≥ 3 times the normal levels associated with bilirubin values ≥ 2 times the normal levels; according to INPULSIS and other clinical trials, increased liver enzymes were reversible and were not associated with liver disease [13]. Cases of drug-induced liver damage, including severe or fatal cases, were predominantly reported within the first three months of treatment. A slightly higher frequency of bleeding in patients treated with Nintedanib was observed in several clinical studies. Epistaxis, which is not severe, was more commonly reported, while severe bleeding events were rarer. However, real-life observation of a group of patients concurrently taking anticoagulants did not reveal more bleeding events [22]. 

An interesting Italian study conducted by Di Battista et al. compared Nintedanib side effects in different populations (IPF vs. SSc) and found no particular differences in tolerance and the ability to manage side effects in the two groups; in SSc patients, however, a shorter time lapse from the first dose to eventual reduction or discontinuation was noted [21]. A real-life multicenter study investigated functional deterioration after Nintedanib dose reduction or discontinuation, confirming the serious impact of the suspension on pulmonary function, suggesting the importance of patient monitoring and symptomatic treatment of AEs rather than discontinuing antifibrosant therapy [23]. In contrast, a temporary reduction or suspension of the dosage did not seem to affect the progression of the disease in a short-term follow up [25].

Regarding Pirfenidone, adverse events (AEs) described with a frequency > 5% at a dose of 2403 mg/day were nausea, dyspepsia, vomiting, anorexia, asthenia, skin rashes, photosensitization, and dizziness.

ASCEND and CAPACITY studies registered 15% of patients who discontinued treatment due to AEs (vs. 9% in the placebo group); in particular, 1% of patients interrupted Pirfenidone for skin rash or nausea. Furthermore, gastrointestinal tract-related AEs and photosensitization were observed to be dose related and easily reduced by taking Pirfenidone with food for the first time and avoiding sun exposure or using adequate sunscreen for the second [13,14,15,16,17,26]. Finally, laboratory abnormalities such as hyperglycemia, hyponatremia, hypophosphatemia, and transaminase alterations (values of up to 3 times the normal range) have been described, all of which are reversible and without clinical sequelae. The permanent discontinuation of Pirfenidone is necessary for transaminase values > 3 to <5 times the upper limit of normal (ULN) with hyperbilirubinemia or signs and symptoms of liver damage. This measure is also mandatory in cases of transaminase levels ≥ 5 times the ULN. 

For the risk of hepatotoxicity associated with both drugs, measuring transaminases and bilirubin before initiating treatment, during the first month, and at regular intervals thereafter is recommended. 

It was highlighted that most of the ADRs leading to treatment discontinuation usually occur in the first 6 months of therapy for both drugs, but while patients tolerating a full dose of Pirfenidone along this time would likely tolerate it thereafter, tolerance to Nintedanib varies, even for a long time after initiation, and drug reduction may be necessary at any time. This strategy usually allows the drug to be tolerated and treatment to continue, resulting in a lower percentage of patients having to discontinue therapy. Importantly, the dose reduction made to manage ADRs seems to not reduce the benefits of treatment in decreasing lung function decline [28]. 

## 4. Cost-Effective Analysis of Antifibrotic Drugs

The cost of antifibrosant therapies depends on several factors, including the dosage of the drug, the duration of therapy, and the country in which the drug is prescribed. There are several studies in the literature that have analyzed the cost-effectiveness of Pirfenidone and Nintedanib, both in Europe and the US, and others that have relied on mathematical models to predict the cost of antifibrosant therapy in the long term. For example, in the United Kingdom (UK), the yearly listed price for Pirfenidone equates to USD 36,070.80. Likewise, in Belgium, the annual listed price for Nintedanib is approximately USD 28,910 [104]. In the US, a 2021 antifibrosant drug adoption study by Dempsey et al. estimated a mean cost per month of USD 397.51 for Nintedanib and a mean cost per month of USD 394.49 for Pirfenidone [105]. Westernick et al. demonstrated that in patients with fibrosing interstitial lung disease, the comparison of Nintedanib treatment to placebo, in addition to standard care, yielded an incremental cost-effectiveness ratio of EUR 60,690 per quality-adjusted life year over a decade; so, Nintedanib treatment could offer significant health improvements for patients while remaining cost-effective within the typical willingness-to-pay threshold in the Netherlands [106]. 

Regarding the comparison between the two drugs, a Belgian study found Nintedanib to be more cost-effective than Pirfenidone [107]. Demonstrating that the cost-effectiveness analysis differs greatly from country to country, an analysis was recently conducted in the United States by Dempsey et al. found that antifibrotic medications cost more than USD 110,000 per year compared to USD 12,291 annually for symptom management and, in particular, Pirfenidone was found to be more expensive than Nintedanib [104].

Beyond these data, a study was conducted in 2023 by Lokke et al. on the burden of Disease and Productivity Loss in Europe in patients with ILD, which showed that this disease represents a significant burden on society. The analysis was conducted particularly in terms of annual sick days, early retirement, loss of work, and permanent disability, and affect not only the patients but also impact caregivers’ quality of life across various dimensions, including sleep and health, daily activities, emotional well-being, social interactions, and financial aspects; thus demonstrating that it is crucial to minimize the impact of the disease on productivity [106,107,108].

## 5. Discussion

Safety profiles of both Pirfenidone and Nintedanib in patients with IPF have been widely debated over many clinical trials [13,14,15,16,17,18,19,20,21,22,23,24,25,26,27,28]. The majority of AEs concerning both antifibrotic drugs were defined as mild to moderate (especially mild), without any long-term sequela or any case of death possibly linked to these two drugs. In addition, AEs were, in almost all the patients, easily manageable with therapy adjustments or additional treatments for the symptom; for example, the administration of anti-diarrheals and probiotics seemed to increase Nintedanib therapy tolerance in patients who had diarrhea as a main side effect [25]. These two drugs potentially have similar efficacy in slowing down IPF progression, while adverse events are different, and the main reasons that lead patients to permanently stop the therapy may depend on which drug is chosen [26,27,28]. In Pirfenidone treatment cohorts, it has been highlighted that firstly, skin adverse events, especially photosensitivity reactions, and secondly, gastrointestinal intolerance (mainly nausea), are the main AEs in Pirfenidone cohorts and most likely led to therapy adjustments, as shown in Table 1. 

In addition, it seems that skin AEs are more likely to lead to a permanent interruption of therapy, which may be justified by the fact that skin reactions are less prone to be managed by therapy adjustments or additional treatments, or tolerated by the patients [28]. 

Instead, in Nintedanib treatment cohorts, it has been shown that gastrointestinal adverse events are widely the main reason for discontinuation, interruption, and dose reduction in treatment, while cutaneous adverse events were never experienced. After analyzing the main clinical trial that aimed to investigate the tolerance and safety of Nintedanib it has defined diarrhea as the main reason for therapy adjustments, as well as the most common AEs in Nintedanib cohorts, as shown in Table 2. 

As expected, there were differences concerning AEs between these two drugs. Information on therapy adjustments and safety profiles of both antifibrotic drugs was exhaustive with regard to IPF patients, but only little data about the other diseases evaluated were available. The discontinuation rates for both antifibrotics drugs vary among all the studies, and this variability may be due in part to several different patients’ tolerance to AEs. Side effects had basically the same rates and were mainly mild to moderate, where diarrhea and nausea were predominant in Nintedanib treatments and skin reactions; nausea and decreased weight were predominant in Pirfenidone treatment. Concerning Pirfenidone, the inadequate response to sunscreen in photosensitive skin reactions was the main trigger for dose interruption, whereas, in Nintedanib, the worst episodes of diarrhea were the main triggers for therapy interruptions. In the future, it may be useful to better stratify the types of patients who should be more tolerant of the use of an antifibrotic drug than the other. This may also help ongoing trials to better understand how to best manage AEs in order to have higher rates of full dosage administration treatments, particularly without any need for therapy discontinuation. 

The various exposed sections highlight that both antifibrotic drugs are valuable in slowing the active fibrosing pathological process of the lung, but none of them induce its regression (Table 3). 

Furthermore, their pharmacodynamic properties only partially target the common resulting pathways of various insults, without modifying the root cause or other aspects of the diseases associated with lung fibrosis, such as exacerbations in IPF or skin alterations in SSc-ILD. Regarding their efficacy, the most satisfactory data, including mortality risk reduction—best predicted by FVC—stem from well-established clinical trials in IPF. Additionally, positive results regarding mortality reduction have also been observed in trials involving PPF and UIP-ILD, distinct from IPF.

Efficacy in SSc-ILD has been proven for Nintedanib, but not yet for Pirfenidone, so while the first one is approved for treatment in different countries, the second one needs further studies for more significant data, which could be of interest in providing a therapeutic alternative for patients who have not tolerated Nintedanib. Finally, only a few studies concern the efficacy of Nintedanib and Pirfenidone in recent COVID-19-related fibrosis. They examine different outcomes—on a relatively small and non-homogeneous population—that support the positive effect of their introduction in therapy; but, due to the high prevalence of this new entity, further efficacy studies are required. The introduction of antifibrotics in the treatment regimen is evaluated considering the progression rate of the fibrosing process and the risk of side effects. The first one is shown by periodic pathophysiological tests and radiological imaging, the latter are well-known and regularly checked by interviews and blood analyses, and they are rarely so dangerous that the immediate withdrawal of the drug is mandatory. In risk–benefit balance, the opportunity of tapering and interruption of steroids and immunosuppressants is important, so the combination between Nintedanib and Pirfenidone due to their different pharmacodynamics or their separate association with drugs of standard protocols is especially interesting, but data on safety, tolerability, and superiority over standard treatment to justify alternative protocols or early introduction are still missing. Regarding the efficacy of antifibrotic drugs in the early treatment of SSc-ILD and post-COVID-ILD, the short time from the radiological detection of lung alterations and the therapy may be the key factor for better outcomes, as ill patients are already monitored for lung involvement.

Current ATS/ERS/JRS/ALAT guidelines for clinical practice in PPF-ILD differ from IPF for the conditional recommendation of Nintedanib when standard therapy fails, where its effect varies from case to case, probably depending on the different ILDs; the recommendation of Pirfenidone is also conditional due to lack of significant data [89]. 

The importance of antifibrotics as possible drugs in SSc-ILD is highlighted in the last American College of Rheumatology guidelines, which strongly recommend against glucocorticoids (GC) as first-line ILD treatment in SSc-ILD but conditionally recommend Nintedanib as a first-line ILD treatment option, and there was no consensus on the intravenous use of GC in rapidly progressive ILDs as evidence of a renal crisis [90]. 

As reported by the recent review by Alrajhi, antifibrotics can be the first therapy of choice in post-COVID-ILD with the UIP pattern (less frequent than OP and NSIP) and are indicated by a progressive fibrotic type not responding to immunosuppressants [63]. PASC fibrotic alterations were observed as early as three weeks after the infection of severely ill hospitalized patients [91]. It is reasonable to assume that the timely positive effect of antifibrotics depended not only on their antifibrotic activity but also on their anti-inflammatory and antioxidant properties; for example, pharmacodynamic murine studies had already shown that Pirfenidone can significantly ameliorate fibrosis and ARDS-induced fibrosis in vitro when started early in the course of the disease [92]. 

Early antifibrotic treatment starting 3 weeks from the onset of symptoms is controversial; there is not enough literature to establish if it does prevent and stop the fibrosis progression, and long-term studies monitoring the progression of early-treated patients are lacking [93]. 

The complexity of drawing generalizable conclusions about post-COVID-19 interstitial lung disease (PCILD) is compounded by real-world studies conducted during different pandemic waves, with diverse management approaches and varying follow-up periods. Further complicating matters is the lack of a standardized definition of PCILD, as evidenced by significant heterogeneity in meta-analyses on the subject [94,95]. 

The natural history of fibrosing ILD after acute severe COVID-19 is evolving, as well as its clinical significance. Furthermore, COVID-ILD UIP fibrosing patterns and IPF share clinical and biological similarities, which support the inclusion of PACS fibrosis in PPF-ILDs [61,93,94], even if the systematic review by Gupta et al. about the incidence and prevalence of ILDs does not specifically mention this emerging fibrosis [98]. 

The above-mentioned alarmingly high post-COVID-19 fibrosis estimated prevalence—approximately 30 times higher than the prevalence of IPF—as well as recent studies suggesting that this tremendous increase in lung parenchymal distortion may ultimately impact up to eleven percent of patients after hospitalization with COVID-19, support the need for validated treatment protocols to contrast the progression of lung alterations and the emerging consequences of the pandemic, especially in younger patients [61,99,100].

On the other hand, the compared IPF prevalence is probably underestimated because this latter disease is chronic and rare, whilst COVID-19 is an acute and recent pandemic. In the “state of the art for 2023” by Podolanczuk et al., the last epidemiology data report IPF incidence ranging from 1 to 13 per 100,000 persons, and its prevalence is from 3 to 45 per 100,000, specifying the scarcity of reports from different countries [101].

In general, epidemiological data for both idiopathic and non-idiopathic progressive fibrosis indicate a global increase. However, due to differences in reporting, case definitions, and study methodologies, current estimates are likely lower than actual figures. Furthermore, recent redefinitions of various conditions complicate the attribution of a definitive diagnosis, and changes over time in diagnostic criteria prevent the ascertainment of more precise epidemiological data [3,102,103]. In the end, for post-COVID-19 fibrosis, there is a lack of definitive epidemiological data, and the estimate of its prevalence being 30 times higher than IPF could be compromised by overdiagnosis.

Enhancing our understanding of the real burden of PPF is crucial for effective health service planning, particularly in light of the emergence of new antifibrotic treatments, but for that purpose, it would be necessary not only to employ national registries referring to updated and standardized diagnostic criteria—a desirable objective within a reasonable timeframe—but also for patients all over the world to have the same opportunity to receive a diagnosis—an ideal scenario. 

## 6. Conclusions

Antifibrotic drugs are currently included in different protocols for progressive fibrosis management to modify disease behavior. Their side effects are well-known for previous experience in IPF patients, where their effectiveness has been proven. The efficacy of alternative drug associations and the best time of introducing antifibrotic therapy are under investigation. In post-COVID-19 fibrosing ILDs, the efficacy of both Pirfenidone and Nintedanib is supported by theoretical research and different case reports and series, but there is a lack of large long-term trials, and high-quality effectiveness data are yet to come. However, further research is needed to optimize the use of antifibrotic agents in patients with PF-ILDs, which could slow disease progression and decrease all-cause mortality. Finally, other studies are requested to establish the treatments that can stop ILD progression.

## Figures and Tables

**Table 1:** Frequency of AEs with Pirfenidone treatment.

Articles	Pop (n)	Eventual Required Therapy Adjustments	Patients with AEs (%)	Main AEs Documented (%)		
				Skin AEs (%)	Gastrointestinal AEs (%)	Other AEs (%)
Bargagli E. et al. [13]	52 IPF patients	Pirfenidone therapy was well tolerated overall.	_	Cutaneous rash 19%	Dyspepsia and nausea 35%, diarrhea 28%	_
Vancheri C. et al. [14]	379 IPF patients	In nine patients, AEs led to the discontinuation of therapy.	39.3%	14% in all. Photosensitivity reaction 5%, cutaneous rash 3.7%, erythema 3.2%	12.4% in all. Nausea 3.7%, diarrhea 2.1%, dyspepsia 2.1%	_
Jouneau S. et al. [15]	192 IPF patients	32.3% needed a dose reduction, while 17.7% underwent dose interruption. 31.8% needed discontinuation of therapy.	80.1%	Cutaneous rash 12%, photosensitivity reaction 10.9%, erythema 7.8%. Serious ADRs were mostly related to skin ADRs (3.1%)	Decreased weight 29.7%, decreased appetite 18.8%, nausea 15.6%	Fatigue 15.6%
Vietri L. et al. [16]	91 IPF patients	Four patients had to permanently discontinue therapy due to severe photosensitivity reactions. 3.4% needed dose adjustment.	27%	Cutaneous rash 6.5%, photosensitivity reaction 4.3%	Decreased weight 5.4%, nausea 4.3%	Hypertransaminasemia 4.3%
Chang C.Y. et al. [17]	50 IPF patients	4% discontinued therapy, while 10% needed dose adjustment.	52%	28% in all	32% in all.	7% in all
Cameli et al. [26]	134 IPF patients	Fourteen patients permanently discontinued therapy.	31%	Cutaneous rash 13.6%, photosensitivity reaction 11.5%	Decreased weight 17.9%, nausea 7.2%, diarrhea 2.1%	Hypertransaminasemia 5%
Galli J.A. et al. [27]	129 IPF patients	12.4% needed a dose reduction. 20.9% had to discontinue therapy.	_	Photosensitivity reaction 14.7%	Nausea 26.4%, dyspepsia 12.4%	_
Levra S. et al. [28]	192 IPF patients	Forty-five patients underwent a dose reduction, of which fifteen had to temporarily discontinue therapy. Thirty-four patients had to permanently stop administration.	60.4%	Photosensitivity reaction 14.7%, cutaneous rash 6.7%	Nausea 21.9%, decreased weight 12.5%, dyspepsia 11.6%, decreased appetite 8.9%	_
Khanna D. et al. [56]	63 SSc-ILD patients	Nine patients left the study due to AEs and 57.1% had a dose adjustment.	96.8%	Cutaneous rush 17.7%	Nausea 44.4%, gastroesophageal reflux disease 19.0%	Headache 30.2% and fatigue 31.7%
SLS III trial [58]	51 SSc-ILD patients	-	-	Cutaneous rash 20.6%, photosensitivity reaction 6.3%	Nausea 49.2%, vomiting 28.6%	-
Solomon et al. [84]	123 RA-ILD patients	This study is incomplete insofar as its evaluation had to be stopped due to COVID-19 pandemic reasons. Nevertheless, it was rather promising, and no significant differences in side effects were detected regarding RA-ILD patients who underwent Pirfenidone treatment and placebo.				

**Table 2:** Frequency of AEs with Nintedanib treatment.

Articles	Pop (n)	Eventual Required Therapy Adjustments	Patients with AEs (%)	Main AEs Documented (%)		
				Skin AEs (%)	Gastrointestinal AEs (%)	Other AEs (%)
Bargagli E. et al. [13]	30 IPF patients	5% needed treatment interruption. 18% of diarrhea events led to therapy discontinuation.	_	None of these articles have pointed out any major skin reactions to Nintedanib.	Diarrhea 40%.	8% had altered liver enzymes.
Tzouvelekis et al. [18]	94 IPF patients	Twenty patients permanently discontinued therapy.	_		Diarrhea 55.3%	_
Hughes G. et al. [19]	124 IPF patients	26% had to discontinue therapy and 19% needed to permanently stop treatment.	96%, of which 82% had at least two AE events		Diarrhea 24%, nausea 13%, decreased appetite 10%.	Tiredness 9%.
Campochiaro et al. [20]	90 SSc-ILD patients were enrolled, but only 40 patients’ information was available.	10% had to permanently stop therapy.	_		39% in all.	_
Di Battista M. et al. [21]	82 IPF patients	Diarrhea is the main reason for interruption. 64.6% of IPF patients needed a dose reduction and 18.3% had to permanently stop therapy.	72.2% of the IPF group		64.6% in the IPF group had diarrhea, 15.8% had nausea.	17% in the IPF group showed altered liver enzymes.
Di Battista M. et al. [21]	27 SSc-ILD patients	Diarrhea is the main reason for interruption. 70.3% of SSc-ILD patients needed a dose reduction and 11% had to permanently stop therapy.	74% of the SSc-ILD group		66.4% in the SSc-ILD group had diarrhea, 22.2% had nausea.	7.4% in the SSc-ILD group showed altered liver enzymes.
Ruaro B. et al. [22]	56 IPF patients	18% needed a dose reduction, 32% had to discontinue therapy.	_		Gastrointestinal reactions (especially diarrhea) were the main reason for dose reduction and discontinuation.	_
Ruaro B. et al. [23]	54 IPF patients	20.4% had reduced the dosage and 27.8% needed to stop treatment.			Dose reduction and treatment discontinuation were mostly due to gastrointestinal reactions (especially gastrointestinal intolerance and diarrhea).	
Fletcher S.V. et al. [24]	154 IPF patients	44.8% had to discontinue therapy, of which 7.2% discontinued due to diarrhea.	77%		Diarrhea 67.5%, nausea 52.6%, decreased appetite 16.9%.	Six patients had their liver enzymes increased.
Hirasawa Y. et al. [25]	86 IPF patients	28% permanently stopped therapy, of which nine patients stopped due to diarrhea.	58%		Only patients who had diarrhea were enrolled.	_
Cameli et al. [26]	124 IPF patients	Seven patients permanently discontinued therapy.	41.9%		Diarrhea 31.6%, decreased weight 13.7%, nausea 4%.	Hypertransaminasemia 9.6%.
Galli J.A. et al. [27]	57 IPF patients	21.1% underwent a dose reduction, 26.3% discontinued therapy.	_		Diarrhea 52.6%, nausea 29.8%.	_
Levra S. et al. [28]	89 IPF patients	Twenty-seven patients underwent a dose reduction, eleven patients discontinued therapy, and nine patients permanently stopped therapy.	69.6%		Diarrhea 40.8%, nausea 15.8%, weight loss 13.3%.	Hypertransaminasemia 14.2%.
Flaherty KR. et al. [77]	332 patients	Rates of AEs in the Nintedanib group and placebo group were similar overall. Nevertheless, in the Nintedanib group, AEs more frequently led to a permanent dose reduction (33.1%) and treatment interruption (19.6%).	95.5%		Diarrhea 66.9%, nausea 28.9%, vomiting 18.4%, decreased appetite 14.5%.	Nasopharyngitis 13.3, bronchitis 12.3%.

**Table 3:** Major diseases that support the use of Nintedanib and Pirfenidone.

Evaluated Diseases	Why Pirfenidone?	Why Nintedanib?	Global Overview
IPF	It has been shown to slow down disease progression by positively affecting the decline of FVC (which decreases less quickly) [29,30,31,32]. This leads to better outcomes.	As seen with Pirfenidone, patients treated with Nintedanib have shown an impaired decrease in FVC, which has helped to slow down disease progression [37,38,39].	They have shown efficiency, and these two drugs taken isolated or with other therapies did not avoid fatal outcomes in IPF patients.
SSc-ILD	Few valuable clinical trials evaluated the efficacy of Pirfenidone. No studies have shown a benefit of using Pirfenidone in SSc-ILD patients. An improvement with SSc-ILD patient symptoms and HRCT images with Pirfenidone administration [59] was reported.	Many relevant trials have demonstrated that Nintedanib provides significant improvement concerning annual FVC decrease rates, whether taken individually or with MMF. However, the latter administration needs further studies [53,54,55].	For SSc-ILD, additional therapy with Nintedanib is approved in many countries, while Pirfenidone is not. In addition, despite the effectiveness of Nintedanib, it is proven that in SSc patients, gastrointestinal side effects are frequent, which leads to higher rates of treatment interruption.
Post-COVID-19 fibrosis	A study reported significant improvement in lung involvement, assessed by TC, in patients who were administered Pirfenidone than in patients who underwent classic therapy [64].	Several case reports showed that Nintedanib, whether individually or together with steroids, reduced the severity of the disease, reduced the amount of oxygen support required by the patients, and reduced the length of time before the withdrawal of mechanical ventilation [64,71,72].	The use of antifibrotics in post-COVID-19 fibrosis is incredibly poor. Very poor data has been collected concerning this topic, whereas Nintedanib seems to be promising, and Pirfenidone needs more solid further clinical studies.
Sarcoidosis	No data are available, although some clinical trials are starting to be carried out.	Some clinical trials have shown promising data concerning Nintedanib effectiveness in reducing ILD progression in sarcoidosis patients, which is the leading cause of death in the disease.	_
Rheumatoid arthritis (AR)	Its ability to manage oxidative stress and inhibit pro-inflammatory cytokines suggests that Pirfenidone is a valuable future treatment in RA-ILD [80,81]. Indeed, many clinical trials have proven that Pirfenidone treatment has led to better FVC and DLCO parameters [85,86].	RA-ILD patients who underwent Nintedanib treatment showed a clear improvement in their clinical situation compared to the placebo group [75]. Moreover, DLCO parameters and gravity of joint swelling are two more elements that have seen improvements thanks to Nintedanib therapy, although the latter was only investigated in SGK mouse models [81,82].	Both antifibrotic drugs seem to be very effective in RA-ILD. The main pulmonary function test showed important improvement and did not seek any additional or unmanageable adverse events; also, simultaneous treatment with DMARDs did not involve any changes in the effectiveness or safety of both drugs.
Idiopathic inflammatory myopathies (IIM)	Nintedanib therapy showed a significant increase in patients’ survival with subacute ILD symptoms (3 to 6 months). Nintedanib treatment during acute ILD symptoms (less than 3 months) did not show any relevant changes in the survival rate.	Nintedanib therapy has led to slowing down disease development and increased patients’ survival rate [85].	Very poor data have been collected concerning both antifibrotics and IIM-ILD. Few clinical trials tried to start understanding their possible role.
